# Fast Visuomotor Processing of Redundant Targets: The Role of the Right Temporo-Parietal Junction

**DOI:** 10.1371/journal.pone.0002348

**Published:** 2008-06-04

**Authors:** Eric Mooshagian, Jonas Kaplan, Eran Zaidel, Marco Iacoboni

**Affiliations:** 1 Department of Psychology, University of California Los Angeles, Los Angeles, California, United States of America; 2 Brain Research Institute, University of California Los Angeles, Los Angeles, California, United States of America; 3 Ahmanson Lovelace Brain Mapping Center, Department of Psychiatry and Biobehavioral Sciences, Neuropsychiatric Institute, David Geffen School of Medicine at UCLA, University of California Los Angeles, Los Angeles, California, United States of America; University of Southern California, United States of America

## Abstract

Parallel processing of multiple sensory stimuli is critical for efficient, successful interaction with the environment. An experimental approach to studying parallel processing in sensorimotor integration is to examine reaction times to multiple copies of the same stimulus. Reaction times to bilateral copies of light flashes are faster than to single, unilateral light flashes. These faster responses may be due to ‘statistical facilitation’ between independent processing streams engaged by the two copies of the light flash. On some trials, however, reaction times are faster than predicted by statistical facilitation. This indicates that a neural ‘coactivation’ of the two processing streams must have occurred. Here we use fMRI to investigate the neural locus of this coactivation. Subjects responded manually to the detection of unilateral light flashes presented to the left or right visual hemifield, and to the detection of bilateral light flashes. We compared the bilateral trials where subjects' reaction times exceeded the limit predicted by statistical facilitation to bilateral trials that did not exceed the limit. Activity in the right temporo-parietal junction was higher in those bilateral trials that showed coactivation than in those that did not. These results suggest the neural coactivation observed in visuomotor integration occurs at a cognitive rather than sensory or motor stage of processing.

## Introduction

Parallel processing of multiple sensory stimuli is critical for efficient, successful interaction with the environment. It allows for the simultaneous identification of multiple stimuli and thus swift action. An experimental approach to studying parallel processing in sensorimotor integration is to examine responses to multiple copies of the same stimulus. Reaction time tends to be faster to bilateral, redundant copies than to a single copy of a stimulus. Responding faster to redundant stimuli is known as the ‘redundant targets effect’ [1]. The redundant target effect has been obtained with unimodal redundant visual [2] and auditory [3] stimuli as well as with bimodal audio-visual stimuli [4], and it has been observed in both choice [5], and simple detection tasks [2].

Two alternative mechanisms have been proposed to account for the redundant target effect. “Statistical facilitation” posits that the observed facilitation in reaction time to redundant targets occurs because redundant targets activate multiple, independent, parallel processing channels. Each channel processes one of the redundant targets, and its speed varies from trial to trial as a stochastic process. Consequently, reaction time in a given trial reflects the processing time of whichever channel happened to be faster on that trial, causing the average reaction time to multiple redundant targets to be shorter than the average reaction time for any of the two channels alone. By analogy, statistical facilitation is sometimes described as a ‘horse race’, where the winner initiates the response [6]. This mechanism assumes that there is no interaction among the channels. “Coactivation models”, on the other hand, posit that engaging parallel channels results in a multiplicative activation, or interaction of channels, prior to response initiation [7], [8]. This pooled activation, thus, yields a faster initiation of the motor response. The original coactivation model was abstract and did not take into consideration the underlying neural architecture. Recently, however, coactivation has been typically interpreted as indicating neural summation [9]–[14].

Miller [8] proposed a mathematical test (see Methods) to differentiate between statistical facilitation and coactivation accounts of the redundant target effect. His equation establishes the maximum difference between reaction times to single versus redundant presentations for which statistical facilitation can adequately explain the redundant target effect. In practice, this limit is exceeded on some trials, evidence that some other mechanism must be responsible for response facilitation, at least in those trials. [See, for example, 2,3,15,16]. It is important to note that when this limit is exceeded, statistical facilitation is ruled out as an explanation of the redundant target effect. However, when the limit is not exceeded, coactivation cannot be ruled out.

Theoretically, the functional locus of the redundant target effect may occur at a sensory, central (cognitive), or motor stage of processing. The empirical data are mixed. Many studies have ruled out that it occurs at either very early perceptual or late motor stages of processing. The redundant target effect is typically greater with bimodal stimuli (for example, visual-tactile) than with unimodal stimuli (for example, visual-visual) [15]. These instances provide evidence that the effect occurs after early sensory processing, when information from different modalities is integrated (Miller, 1982). On the other hand, two event-related potential studies reported an early locus of the redundant target effect [10], [17]. In both studies, earlier peak P1 latencies were observed for redundant visual stimuli compared to single visual stimulus trials. Another event-related potential study used redundant audio-visual stimuli and reported early audio-visual interactions consistent with sensory processing [18]. Likewise, Cavina-Pratesi et al. [19] addressed whether the redundant target effect occurs as late as a motor stage of processing, using a task where subjects had to withhold responses on trials with stop-signals. Redundant stop-signals were more effective than single stop-signals in inhibiting motor responses. Similarly, responses to redundant stimuli were more difficult to inhibit compared to single stimuli. The effects of redundant signals on motor responses in these two stop-signal experiments suggest that the redundant target effect occurs at a late, pre-motor, stage, prior to late ballistic motor output [19].

As for the anatomical locus of the effect, reports have suggested that it occurs in either extrastriate or premotor regions, in line with information processing accounts of the effect. The event-related potential data suggest that the redundant target effect is detectable in the extrastriate cortex, but the poor spatial resolution of the event-related potential technique makes it difficult to precisely identify sources of influence [20]. A single-trial fMRI study, on the other hand, found increased blood oxygen-level dependent (BOLD) signal in the left and right dorsal premotor cortex and right intraparietal sulcus for redundant compared to single stimulus targets [16]. The premotor activations reported in that study support a later, motor, stage of processing.

Given the conflicting reports in the literature, the critical brain regions associated with parallel processing of stimuli remain a matter of investigation. Importantly, previous studies have only considered redundant versus single target conditions without distinguishing between performance explained by statistical facilitation and coactivation. Therefore an investigation of the neural locus of coactivation must look at these special trials separately.

The bilateral display used in this paradigm introduces an interhemispheric component to the task. Somewhat paradoxically, split brain and acallosal subjects often exhibit redundant target effects much larger than those in normal subjects which often exceed the boundary predicted by statistical facilitation [9], [11], [14]. These results suggest, counterintuitively, a greater degree of interhemispheric interaction in the absence of the corpus callosum and that, in the normal brain, the corpus callosum may serve to inhibit interhemispheric interaction [11]. Analysis of the functional connectivity of brain regions associated with the redundant target effect could prove useful in determining the role of interhemispheric connections in mediating it. Functional connectivity analyses allow us to examine the temporal cross-correlation of brain regions associated with activity in a seed region and are presumed to reflect structural connectivity between functionally related regions [21]. This analysis is complementary to task activation maps because it describes regions that follow the temporal sequence of information processing rather than the regions that engage simultaneously.

In the present study, we used event-related fMRI to investigate the BOLD signal associated specifically with those trials that exceed the limit for the statistical facilitation account of the redundant target effect. Thus, rather than considering the anatomical localization of fast responses to redundant targets in general, we examined the anatomical localization of the neural coactivation. We also used functional connectivity analyses to investigate interaction within and between the hemispheres during instances of coactivation.

## Results

### Behavior

**Figure 1:**
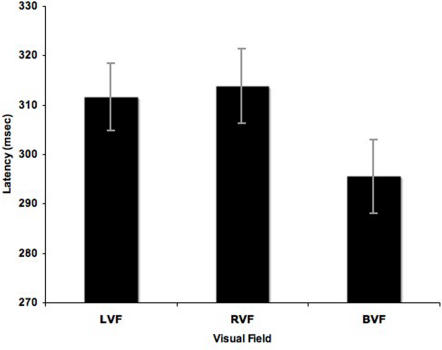
Mean reaction times to each stimulus type. Mean reaction time for unilateral left visual field (LVF), right visual field (RVF) and redundant bilateral visual field (BVF) trials.

There was a main effect of visual field F(2, 28) = 22.012, p = .0001. As predicted, mean reaction time to bilateral trials was significantly faster (295.51±7.42 msec) than mean RT to unilateral left stimuli (311.57±6.79 msec), t(14) = 5.34, p = .0001, or unilateral right stimuli (313.80±7.61 msec), t(14) = 6.08, p = .0001 (Figure 1). The difference in reaction time for bilateral trials and the average of the unilateral trials was 17.18 msec. The Response Hand × Visual Field interaction approached significance, F(2, 28) = 2.678, p = .0862.

**Figure 2:**
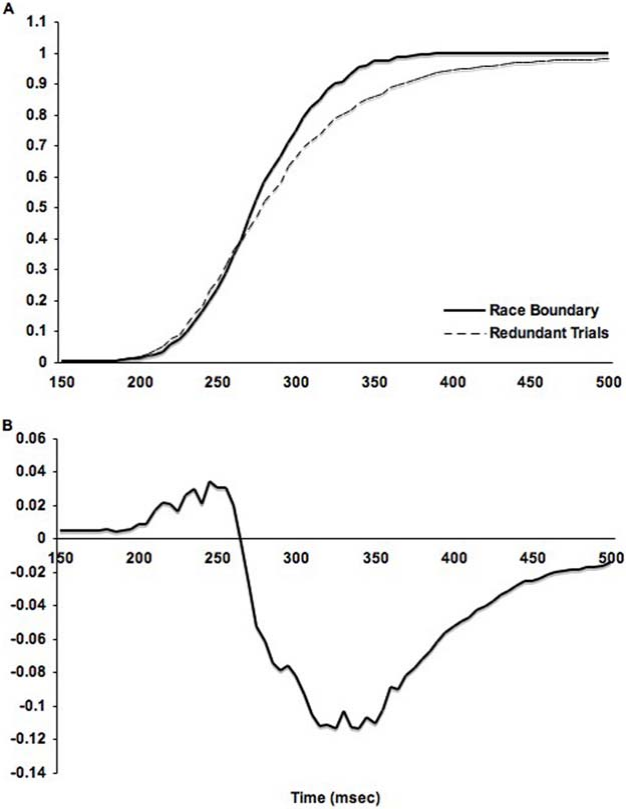
Performance exceeding the limit for statistical facilitation. The top panel shows the cumulative distribution function (CDF) of redundant trials in comparison with the limit for statistical facilitation. Miller's limit is exceeded wherever the redundant trial curve is to the left of the boundary. Redundant trials exceed the statistical facilitation boundary for the fastest reaction times. The bottom panel shows the differences between the CDF for bilateral trials and CDF for the race model inequality.

The fastest trials exceed the limit for statistical facilitation. Figure 2 plots the cumulative distribution function for bilateral trials compared to the sum of unilateral left and unilateral right trials (statistical facilitation boundary). Only reaction time data from those runs in which more than 10% of the bilateral trials exceeded Miller's limit were included so as to match the image analysis (see Brain Imaging Results and Data Processing).

### Functional MRI

The task produced widespread activations throughout the brain, including activations throughout the sensorimotor network. Significant signal changes were found in left and right premotor, supplementary, and primary motor areas as well as the superior parietal lobule, insula, cerebellum and visual cortex.

**Figure 3:**
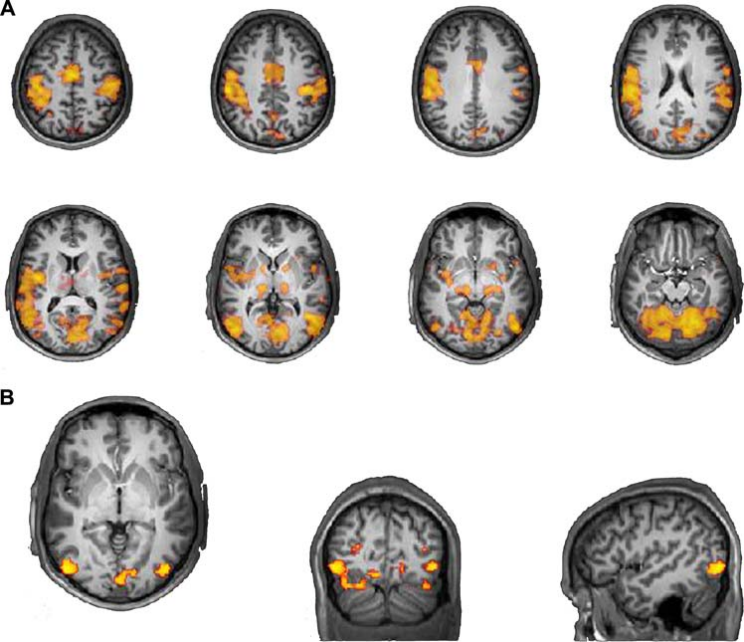
Signal changes for specific contrasts. A Voxels showing significant signal changes in the task compared to rest. B Voxels showing significant signal changes in bilateral minus unilateral VF presentation.

Contrasts of left visual field trials minus right visual field trials, and right visual field trials minus left visual field trials revealed activations in the contralateral visual cortices in line with the lateralized presentation of the stimuli. A contrast of bilateral trials to unilateral trials showed significantly greater signal changes in the visual cortex bilaterally (Figure 3). Activations for each contrast are reported in Table 1.

**Figure 4:**
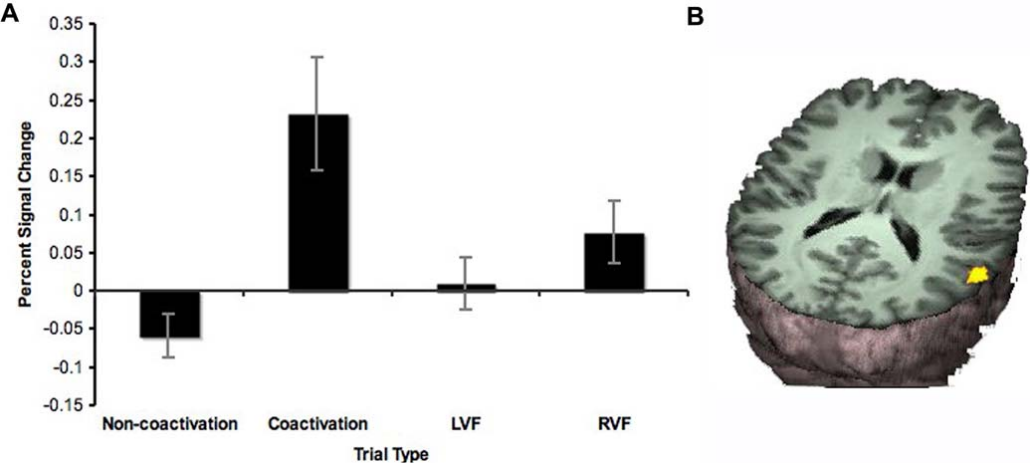
Selective activation of the right temporo-parietal junction during coactivation. A Average percent signal change in the region activated during coactivation compared to non-coactivation trials for each trial type. B Voxels showing significant signal changes during coactivation minus non-coactivation redundant trials.

The comparison of bilateral trials that exceeded the limit to bilateral trials that did not exceed the limit revealed a single region in intersection of the posterior superior temporal gyrus and angular gyrus (right temporo-parietal junction) that showed a significantly higher BOLD signal for the coactivation trials compared to the non-coactivation trials (Figure 4, Table 2). Average percent signal change for each trial type (coactivation, non-coactivation, left visual field, right visual field) in this region revealed a selective increase in BOLD signal for coactivation trials compared to all other trial types. Non-coactivation redundant trials actually revealed a small decrease in percent signal change in the right temporo-parietal junction region (Figure 4).

**Table 1:** MNI coordinates and peak activation statistics for four contrasts.

Contrast	Anatomical Region	Coordinates			Max Z Score
		X	Y	Z	
*Task - Rest*					
	Right postcentral gyrus	48	−18	52	4.77
	Left postcentral gyrus	−58	−24	40	4.93
					5.0
	Left precentral gyrus	−52	−6	40	4.67
	Right postcentral gyrus	46	−22	64	4.63
	Left insula	−42	−8	12	5.02
	SMA	0	−8	50	4.80
	Left mOcG	−52	−72	0	4.76
	Right mOcG	52	−72	−6	4.8
	Right Cerebellum	24	−60	−24	5.18
	Left Cerebellum	−20	−54	−22	4.91
*LVF - RVF*					
	Right Lingual gyrus	18	−82	−12	3.97
*RVF – LVF*					
	Left Fusiform gyrus	−26	−76	−18	5.21
*Bi - Uni*					
	Left mOcG	−50	−78	−2	4.2
	Right mOcG	46	−78	0	3.98
	Right Cuneus	4	−88	4	3.81
	Left Fusiform	−38	−62	16	4.00
	Right Fusiform	30	−62	−18	3.65

MFG = middle frontal gyrus; IOcG = inferior occipital gyrus; OcG = occipital gyrus; STG = superior temporal gyrus.

**Table 2:** MNI coordinates and peak activation for coactivation minus non-coactivation contrast.

Contrast	Anatomical Region	Coordinates			Max Z Score	No. Voxels
		X	Y	Z		
*Coactivation – Non-coactivation*						
	Right AnG	62	−52	18	4.03	75

AnG = angular gyrus

**Figure 5:**
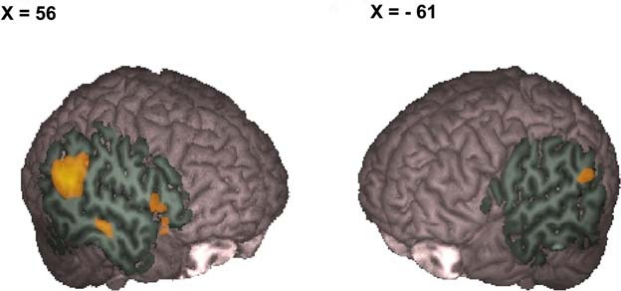
Activity correlated with time-series of the right temporo-parietal region. Voxels showing correlated activity with time-series of the right temporo-parietal region activated in coactivation minus non-coactivation trials as a seed region.

Functional connectivity analysis using the activated right temporo-parietal junction region as a seed revealed a functionally related network that includes the left temporo-parietal junction, right inferior frontal gyrus, and right middle temporal gyrus (Figure 5, Table 3).

**Table 3:** MNI coordinates and peak activations for functional connectivity analysis using timeseries of the right superior temporal gyrus as a seed region.

Contrast	Anatomical Region	Coordinates			Max Z Score
		X	Y	Z	
	Right AnG	62	−52	18	13.503
	Midline Parietal/Occipital junction	−2	−74	34	9.66
	Left AnG	−62	−58	20	9.10
	Right MTG	56	−26	−12	9.33
	Right MFG	48	30	28	8.84
	Right IFG	54	12	8	8.79
	Right CG	6	34	26	8.70
	Right CG	4	−22	40	8.55
	Right cuneus	14	−72	34	8.48
	Right frontal pole	26	56	18	8.45
	Right temporal pole	50	18	−12	8.43
	Right SFG	8	54	22	8.29
	Right MFG	40	14	54	8.27
	Right precuneus	6	−54	32	8.25
	Right temporal pole	44	16	−30	8.10
	Right thalamus	10	−18	−2	8.10
	Right SFG	4	30	46	8.10
	Right ITG	48	−46	−22	8.01

AnG = angular gyrus; CG = cingulate gyrus; IFG = inferior frontal gyrus; IFG = inferior frontal gyrus; ITG = inferior temporal gyrus; MFG = middle frontal gyrus; MTG = middle temporal gyrus; SFG = superior frontal gyrus; OcG = Occiptial gyrus

## Discussion

Our main objective in the present study was to shed light on the functional and neuroanatomical loci associated with coactivation of multiple channels of sensory processing. To the best of our knowledge, this is the first fMRI study to report BOLD activity specifically for bilateral, redundant targets on which performance exceeds the limit predicted by statistical facilitation. Performance that exceeds this limit can only be explained by coactivation. We reasoned that it could be profitable to focus on these trials as they provide clear instances of coactivation occurring. Thus, our imaging analyses focused on the BOLD activity of trials in which performance exceeded the limit predicted by statistical facilitation.

Using this novel approach, we observed selective increase in BOLD signal centered in the right angular gyrus (temporo-parietal junction) for coactivation compared to non-coactivation redundant target trials. Recall that these trials are visually identical yet on some trials, the speeds of responses are so fast that they exceed the upper limit of statistical facilitation. Our results fit well with the larger body of work on the redundant target effect which concluded that the functional locus of the redundant target effect is post-perceptual, but prior to ballistic motor output [10], [16], [19].

The right temporo-parietal junction is an established sensorimotor region important for spatial attention. From an information processing point of view, the right temporo-parietal junction is situated in a central ‘cognitive’ stage of processing. Damage in the region is associated with visuospatial neglect [22], [23] and transcranial magnetic stimulation induces hemiextinction when applied over the right temporo-parietal junction [24]. In terms of functional anatomy, the right temporo-parietal junction has been implicated as part of a larger right-lateralized ventral fronto-parietal system that also includes the middle and inferior frontal gyri and is activated during detection of behaviorally relevant, salient, unattended stimuli [25]. Indeed, our functional connectivity analyses revealed activity in the homologous, albeit a more restricted temporo-parietal junction region in the left hemisphere as well as the right middle temporal gyrus, right middle frontal gyrus and right inferior frontal, largely consistent with the proposed network.

The question occurs whether there exist trials where coactivation occurs, but which do not exceed the limit for statistical facilitation. Figure 4 suggests that the answer is “no”, at least in the right temporo-parietal junction. Non-coactivation trials do not activate this region at all (relative to rest). This indicates that the right temporo-parietal junction is active only during coactivation trials that exceed the limit for statistical facilitation. However, our data cannot exclude that coactivation may occur in other brain regions for trials not exceeding the limit of statistical facilitation.

**Figure 6:**
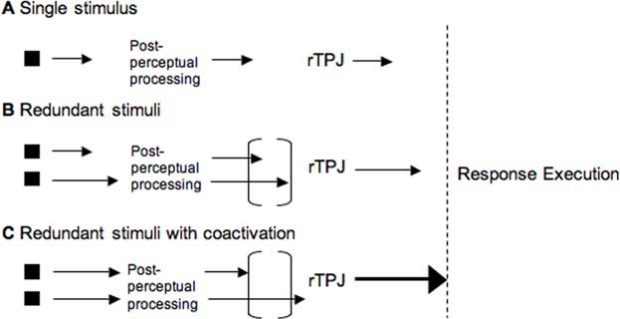
A schematic model of how reaction time differences under single target, non-coactivation, and coactivation instances, occur. In the single target condition A, the reaction time is determined by the speed of processing of the single stimulus. In the redundant target condition B, the reaction time is determined by a race between two independent and equal processing channels. The faster of the two channels determines the speed of the response. However, the delay between the two channels falls inside a window (indicated by the square brackets) for which no coactivation occurs. A longer arrow represents a faster channel. In the special redundant target case when Miller's limit is exceeded C, the two processing channels operate at just the right delay (see text for full explanation) resulting in a difference that exceeds the critical window and thus a coactivation at the right temporo-parietal junction (rTPJ). The coactivation of this region by the two processing channels results in threshold for firing being reached faster and consequently faster processing downstream of the right TPJ, ultimately resulting in reaction times that are faster than in either the single stimulus or redundant target conditions. The threshold for response execution is represented by the vertical dashed line.

How do identical stimuli result in such differences in reaction time? A schematic model of how this might occur is presented (Figure 6). Under single target conditions, the reaction time is determined by the processing speed of a single activated channel (Figure 6A). However, in redundant bilateral targets conditions, one of two possible scenarios occurs. In the typical redundant targets case, a statistical facilitation occurs where the faster of two independent processing streams determines the speed of response, resulting in faster average reaction times compared to the single target conditions (Figure 6B). On some redundant trials, however, a reaction time advantage beyond that predicted by statistical facilitation occurs (coactivation). On these trials, the two parallel processing streams operate at just the right delay for coactivation (Figure 6C), as suggested by previous data on callosal patients [14]. This would be due to intrinsic properties of oscillatory systems, as cortical neural systems tend to be (see ref. 14 for a full explanation of the model.) The probability of such a delay is much greater in the split-brain than in the normal brain.

Split-brain patients often exhibit a redundant target effect that exceeds statistical facilitation [9], [11], [14], [26]. Reuter-Lorenz et al. (1995) proposed an ‘and-or’ model to explain the paradoxically enhanced redundant target effect in simple reaction time that exceeds statistical facilitation observed in split-brain patients. They posited that coactivation occurs at a response selection stage and acts as an ‘and’ gate that requires input from both hemispheres. Under this scheme, a redundant target effect is due to the release of a chronic inhibition on motor pathways under bilateral redundant stimuli conditions. This model was ruled out on empirical grounds [14]. Corballis et al. [26] offered an alternative account where the corpus callosum normally serves to inhibit interhemispheric signals, while in the split brain, interhemispheric inhibition is released. Thus, in the healthy brain, performance that exceeds the limit is rare and intermittent, presumably due to removal of inhibition during rare and limited fluctuations in interhemispheric inhibition. By contrast, in the split brain, this left hemisphere inhibition is constantly released because the corpus callosum is sectioned, resulting in the paradoxical ‘hyper’ redundant target effect.

Our own data showing the right lateralized activation for reaction times that exceed the statistical facilitation limit and the right lateralized functional network observed here for bilateral trials indeed suggest a role for the corpus callosum in mediating the redundant target effect. The dynamic modulation of interhemispheric inhibition may account for why healthy subjects' performance is typically explained by statistical facilitation, while there are also infrequent, yet clear, instances of coactivation in the healthy brain.

A few studies have investigated the redundant target effect in visuomotor integration of redundant stimuli using fMRI. However, even though these studies used the same general paradigm, they investigated different aspects of the visuomotor transformations required by the task. For instance, Iacoboni et al. [14] studied individual differences in the redundant target effect in patients with callosal agenesis. They interpreted their findings as suggesting different forms of cortico-subcortical interactions in callosal agenesis patients with and without performance that exceeds statistical facilitation. Iacoboni & Zaidel [16] used single-trial fMRI to look at the neural correlates of the redundant target effect in the healthy brain. Their results revealed increased BOLD signal for responses to bilaterally redundant stimuli compared to unilateral single stimuli in the precentral gyri bilaterally, left postcentral gyrus, and right intraparietal sulcus. Here, we investigated yet another aspect of the performance at this task, namely those instances of coactivation in the healthy brain. Different brain regions seem associated with these different aspects of the performance at the task, suggesting a fair amount of regional specialization for the visuomotor transformations that occur.

Taken together, our data suggest that a right hemisphere ventrolateral network encompassing the temporo-parietal junction and the inferior frontal cortex is responsible for the transient, very rapid parallel processing of visuomotor information. It is unclear whether the transient nature of this rapid visuomotor processing is due to waxing and waning of activation in the right ventrolateral network or to fluctuating inhibition from the left hemisphere.

## Materials and Methods

### Subjects

Fifteen right-handed subjects (8 male, 7 female) were recruited and compensated for their participation. Subjects gave written informed consent according to the guidelines of the UCLA Institutional Review Board. The UCLA Institutional Review Board approved all aspects of the study. All participants were screened to rule out medication use, head trauma, and history of neurological or psychiatric disorders, substance abuse, or other serious medical conditions.

### Behavioral Task

The software program Presentation® (www.neurobs.com) was used to present stimuli and record latency data. Visual stimuli were presented through magnet-compatible goggles (Resonance Technology, Inc.). A central fixation cross was displayed during the entire experiment. On each trial, subjects saw a briefly presented white box against a black background. The stimuli subtended 1.0 degree of visual angle and were 5 centimeters from the fixation cross to the center of the stimulus. The stimulus was presented for 50 msec following a random interval between 250–1000 msec. A random interval was used to prevent anticipatory responses in the detection task. Stimuli were presented in either the left or the right visual field (‘unilateral’ condition), or in both visual fields simultaneously (‘redundant’ condition). For all trials, the subject's task was to respond as quickly and accurately as possible after detecting the light stimulus regardless of stimulus location by pressing a response button.

Subjects completed 4 functional runs of 114 trials each, comprising equal numbers of left visual field, right visual field, and bilateral visual field stimulus presentations. The three trial types were intermixed in an order optimized to produce maximal signal discriminability and to ensure temporal jitter among the three categories using Optseq2 (http://surfer.nmr.mgh.harvard.edu/optseq). Subjects responded via a response box situated on their torso while in a supine position in the scanner. Responses were made with the left or right index finger, and response hand was counterbalanced across run.

### Functional MRI Acquisition

Brain images were acquired using a Siemens Allegra 3.0 T MRI scanner. Two sets of high-resolution anatomical brain images were acquired for registration purposes. We acquired an MP-RAGE structural volume (TR = 2300, TE = 2.93, flip angle = 8°) with 160 sagittal slices, each 1 mm thick with .5 mm gap and 1.33 mm×1.33 mm in-plane resolution. We also acquired a T2-weighted co-planar volume (TR = 5000, TE = 33, flip angle = 90°) with 36 transverse slices covering the whole brain, each 3 mm thick with 1 mm gap, a 128×128 matrix and an in-plane resolution of 1.5 mm×1.5 mm.

Each functional run involved the acquisition of 156 EPI volumes (gradient-echo, TR = 2000, TE = 25, flip angle = 90°), each with 36 transverse slices, 3 mm thick, 1 mm gap, and a 64×64 matrix yielding an in-plane resolution of 3 mm×3 mm. A functional run lasted 5 minutes and 12 seconds, and each subject completed 4 functional runs.

### Data Processing and Statistical Analysis

#### RT Analysis

Response time data were submitted to repeated-measures ANOVA with Response Hand (left, right) and Visual Field (bilateral, left, right) as within-subject variables. The redundancy gain was computed by subtracting the median RT for bilateral trials from the median RT for unilateral trials.

To distinguish between statistical facilitation and coactivation we used the approach described by Miller [7]. The “[horse] race model inequality” (Equation 1) establishes the upper limit which statistical facilitation can reach:
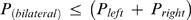
(1)In equation 1, the limit for statistical facilitation can be determined by summing the rank ordered RTs (cumulative distribution functions, CDFs) for the two single stimulus conditions (left and right visual field). The left side of the equation indicates that the fastest responses to redundant stimuli are faster than the fastest single stimulus trials. When this occurs, statistical facilitation cannot adequately explain the redundant target effect and performance reflects the occurrence of coactivation.

To evaluate the inequality, we proceeded as follows. We rank-ordered RTs from fastest to slowest for each stimulus type by subject. We used the resulting cumulative distribution functions (CDFs) from each subject to compute the average CDF for each stimulus type. All the RTs from each subject were averaged at each point in the rank order for each stimulus type. We summed the CDFs for the unilateral left and right trials. The summed CDF for the unilateral trials was then compared to the CDF of the bilaterally presented trials. Probability models require that the CDF of for bilateral trials be everywhere to the right of the summed CDFs of the unilateral trials (Figure 2). When the CDF for bilateral trials is to the left of the CDF for the unilateral trials, coactivation occurs.

#### Functional MRI Analysis

Analysis was carried out using FEAT (FMRI Expert Analysis Tool), part of FSL (FMRIB's Software Library, www.fmrib.ox.ac.uk/fsl). After motion correction, images were temporally high-pass filtered with a cutoff period of 50 seconds and smoothed using an 8 mm Gaussian FHWM algorithm in 3 dimensions.

We modeled the BOLD response using a separate explanatory variable (EV) for each of the three stimulus types (left visual field, right visual field, and bilateral). For each stimulus type, the design was convolved with a gamma function to produce an expected BOLD response. The temporal derivative of this timecourse was also included in the model for each EV. Functional data were then fitted to the model using FSL's implementation of the general linear model.

Each subject's statistical data were then warped into a standard space based on the MNI-152 atlas. We used FLIRT to register the functional data to the atlas space in three stages. First, functional images were aligned with the high-resolution co-planar T2-weighted image using a 6 degrees of freedom rigid-body warping procedure. Next, the co-planar volume was registered to the T1-weighted MP-RAGE using a 6 degrees of freedom rigid-body warp. Finally, the MP-RAGE was registered to the standard MNI atlas with a 12 degrees of freedom affine transformation.

After analyzing the functional data for each subject, data were passed into a higher-level mixed effects analysis. Higher-level analysis was carried out using FLAME (FMRIB's Local Analysis of Mixed Effects) [27]. Z (Gaussianised T/F) statistic images were thresholded using clusters determined by Z>2.3, uncorrected.

In order to examine brain activity involved in coactivation, we ran another analysis that reclassified each bilateral trial as either a coactivation trial or as a non-coactivation trial. For each run, we compared the CDF for redundant (bilateral) trials to the CDF for unilateral trials. Any trials falling within the range of the CDF that exceeded the limit established for statistical facilitation were considered coactivation trials. In this analysis, there were four EVs at the subject level: right visual field trials, left visual field trials, coactivation, and non-coactivation trials. The analysis of coactivation trials included only those runs in which >10% of responses to bilateral trials exceeded the race model inequality. 25 of 60 total runs were excluded for not meeting these criteria.

Coactivation trials are faster than non-coactivation trials by definition. To exclude the possibility that the physiological differences between coactivation and non-coactivation bilateral trials were due simply to random fluctuations in speed, we compared BOLD response on the fastest and slowest 20% of unilateral trials. The analysis of fast versus slow unilateral trials did not show any significant differences in BOLD activation, arguing against a simple speed account.

For the functional connectivity analysis, we created a “seed” ROI based on the right temporo-parietal region activated during coactivation trials from our group-level analysis. The seed mask was warped into each subject's native space and used to extract a timeseries by averaging across all voxels within the mask. We then carried out multiple regression analysis using the seed timeseries as a regressor to identify voxels that were correlated with the activity within the ROI. This produced subject-level maps of all active and deactivated voxels associated with the timeseries regressor. Group-level analyses were carried out using a mixed-effects model implemented in FSL and produced thresholded z-score maps of functional connectivity.
